# Nematicidal Metabolites from the Actinomycete *Micromonospora* sp. WH06

**DOI:** 10.3390/microorganisms10112274

**Published:** 2022-11-16

**Authors:** Yuan Ran, Yu Zhang, Xin Wang, Guohong Li

**Affiliations:** State Key Laboratory for Conservation and Utilization of Bio-Resources in Yunnan, Key Laboratory for Southwest Microbial Diversity of the Ministry of Education, Yunnan University, Kunming 650091, China

**Keywords:** actinomycete, *Micromonospora* sp., *Meloidogyne incognita*, metabolites, nematicidal, egg hatching

## Abstract

A nematicidal actinomycete strain WH06 was isolated from soil samples and was identified using 16S rRNA as *Micromonospora* sp. Through medium screening and fermentation, 10 metabolites were isolated from the ethyl acetate extract of its fermentation broth using Sephadex LH-20 and silica gel column chromatography. These compounds were identified as N-acetyltyramine (**1**), N-acetyltryptamine (**2**), 1-methylhydantoin (**3**), benzenepropanoic acid (**4**), cyclo-(L-Pro-L-Tyr) (**5**), cyclo(L-Phe-Gly) (**6**), catechol (**7**), methyl (4-hydroxyphenyl)acetate (**8**), 3-hydroxybenzoic acid (**9**), and 4-hydroxybenzoic acid (**10**). In an in vitro assay against *Meloidogyne incognita*, a root-knot nematode, compounds **1**, **4, 9,** and **10** show nematicidal activity. Among them, benzenepropanoic acid (**4**) causes 99.02% mortality of nematode at 200 μg mL^−1^ after 72 h. Moreover, compound **4** also displays activity in inhibiting egg hatching of *M. incognita*. This suggests that *Micromonospora* sp. WH06 is a promising candidate for biocontrol of *M. incognita*.

## 1. Introduction

Plant parasitic nematodes are a major threat to agriculture. They cause global crop losses of nearly USD 173 billion annually [1]. The root-knot nematodes (*Meloidogyne* spp.) are destructive parasites that severely damage major crops [2] by destroying plant roots [3]. As an aggravating agent for the introduction of bacterial and fungal pathogens, they worsen the situation [4]. *Meloidogyne incognita* is one of the best-known species among the nearly 100 reported species of *Meloidogyne* spp. [5].

Root-knot nematodes are mainly distributed in the soil and attack the roots of plants, making it difficult to develop a suitable control method [6]. Currently, chemical nematicides are mainly used to control nematodes, with synthetic chemicals being phased out due to their potential biological and environmental hazards [4]. The long-term and frequent use of chemical nematicides such as organophosphorus increases the possibility of resistance development by the nematodes [7], and this may lead to enhanced soil biodegradation mechanisms and a lack of efficacy under field conditions [8]. Compared to chemical pesticide controls, microbial pesticides used as biological controls are friendly to plants and animals, humans, and ecosystems, in addition to being easily accessible, as well as favoring biodegradation [9,10]. Soil microorganisms are sources of biological nematicides that stimulate plant growth and that inhibit plant parasitic nematode populations [11]. However, microbial biocontrol agents have their disadvantages. Some of them cannot provide consistent results when applied in field conditions, and because they direct intervention into the ecosystem, it is necessary to evaluate the potential hazards that may ensue from their application [12].

Actinomycetes are one of the most important producers of active metabolites [13], and they are a potential source of many active compounds with antifungal, antitumor, antibacterial, antiviral, antiparasitic, and other properties [14]. *Micromonospora* is one of the most important genera of actinomycetes [15]. Natural metabolites isolated from *Micromonospora* spp. have led to a large number of antibiotics for clinical use, such as gentamycin [16], and their natural products are also used as enzyme inhibitors, antioxidants, antitumor agents, and antiparasitic agents [17]. *Micromonospora* plays an important role in biocontrol, nitrogen fixation, and biofuel production [13]. It was reported that a strain of *Micromonospora* produces antibiotic G-418, with a high degree of activity against protozoa, amoeba, cestode, and pinworm infections in mice [18]. It was also reported that diazepinomicin from *Micromonospora* strain RV115 exhibits antiparasitic activity against trypomastigote forms of *Trypanosoma brucei* with an IC_50_ of 13.5 µM. [19]. The antiparasitic activity of *Micromonospora* has focused only on animal parasites, while little has been reported against root-knot nematodes.

In this study, the strain WH06 was isolated from the soil and was identified as *Micromonospora* sp. Through medium screening, large fermentation, the collection of crude extracts, and purification of the secondary metabolites, 10 compounds were identified. Among them, four compounds, N-acetyltyramine (**1**), benzenepropanoic acid (**4**), 3-hydroxybenzoic acid (**9**), and 4-hydroxybenzoic acid (**10**), exhibit nematicidal activity against *M. incognita*. Meanwhile, compound **4** shows an inhibitory effect on *M. incognita* egg hatching.

## 2. Materials and Methods

### 2.1. General Materials and Experimental Instruments

Soil samples were collected in the Tianshan Mountains, Xinjiang Uygur Autonomous Region, China.

Acquisition of *M. incognita*: Egg masses were collected from infested tomato roots. The nematodes were isolated from the roots by referring to reference [20]. Egg masses were incubated at 28 °C for 1–2 days to obtain juveniles, which were collected, prepared in sterile water, and used as a suspension.

Culture of *Caenorhabditis elegans*: An appropriate amount of oats (25 g) was added to a 250 mL conical flask, with water to just above the oats, and sterilized. After cooling, about 1000 *C. elegans* were added to the oats medium and incubated for one week. The culture method is referred to the literature [21]. The nematodes were gently scraped out along the wall of the flask and washed out in sterile water separated by three layers of microscope paper as a suspension.

Silica gel G (Qingdao Ocean Chemical Company, Qingdao, China), Sephadex LH-20 (Amerson Biosciences, Piscataway, NJ, USA), thin-layer chromatography (TLC) on silica gel plate GF254 (Qingdao Ocean Chemical Company, Qingdao, China).

Electrospray ionization mass spectrometry (ESI–MS) spectra were recorded on a Thermo high-resolution Q Exactive Focus mass spectrometer (Thermo, Bremen, Germany). Nuclear magnetic resonance (NMR) spectra were measured on an Avance III-600 spectrometer (Bruker Biospin, Rheinstetten, Germany).

### 2.2. Media Formulations

Strains isolation, culture, and activity screening media include Gao I medium (GM), glucose yeast malt agar (GYM), nutrient broth (NB), and Luria–Bertani (LB) media.

Fermentation screening media: 1# medium (30 g soluble starch, 10 g glucose, 20 g soybean powder, 5 g peptone, 0.5 g K_2_HPO_4_, 0.5 g MgSO_4_, 1 g CaCO_3_, water 1000 mL, pH = 7.2); 2# medium (2 g soybean powder, 2 g peptone, 20 g glucose, 5 g soluble starch, 2 g yeast paste, 4 g NaCl, 0.5 g K_2_HPO_4_, 0.5 g MgSO_4_, 2 g CaCO_3_, water 1000 mL, pH 7.2–7.4); 3# medium (20 g soybean powder, 20 g mannitol, 2 g CaCO_3_, water 1000 mL, pH 7.2–7.4); 4# medium (20 g glycerol, 5 g beef paste, 5 g yeast paste, 5 g casein, 1 g peptone, 4 g CaCO_3_, water 1000 mL, pH 7.2–7.4), 5# medium (10 g soluble starch, 5 g glucose, 1 g yeast paste, 1.5 g casein, 1 g beef paste, 1 g CaCO_3_, water 1000 mL, pH 7.2–7.4); 6# medium (ISP1) (5 g tryptone, 3 g yeast extract, water 1000 mL, pH 7.2–7.4); 7# medium (ISP2) (4 g yeast extract, 10 g malt extract, 20 g glucose, water 1000 mL, pH 7.2–7.4); 8# medium (10 g glucose, 4 g yeast extract, 4 g peptone, 4 g K_2_HPO_4_, 2 g KH_2_PO_4_, 0.5 g MgSO_4_·7H_2_O, water 1000 mL, pH 7.2–7.4); 9# medium (5 g soluble starch, 2 g yeast extract, 10 g sucrose, 2 g NaCl, 2 g peptone, 0.5 g K_2_HPO_4_, 0.5 g MgSO_4_·7H_2_O, 10 g glucose, 1 g CaCO_3_, 2% soybean powder extract 100 mL, water volume to 1000 mL, pH 7.2–7.4); 10# medium (10 g millet, 10 g glucose, 3 g peptone, 2.5 g NaCl, 2 g CaCO_3_, water 1000 mL, pH 7.2–7.4).

All solid media were supplemented with 20 g L^−1^ agar based on the above liquid media.

### 2.3. Isolation and Activity Screening of Strains

The soil samples were separated using the dilution plate method referred to in the literature [22]. A total of 10 g dry soil samples was added to a test tube containing 90 mL sterile distilled water, shaken for 30 min at 28 °C at 200 rpm. A total of 1 mL of soil suspension was aspirated into 9 mL of sterile distilled water and subjected to a 10-fold gradient dilution to a final concentration of 10^−2^~10^−7^. A 0.1 mL volume of the dilution was aspirated and applied to the GM and GYM media. The plates were cultured at 28 °C for 20 days. Starting from the seventh day, newly emerged colonies were selected and purified on the purification medium every day. After 7 to 10 days of incubation at 28 °C, single colonies were removed from the agar plates and passaged until pure cultures were obtained. Purified strains were stored as glycerol aqueous suspensions (20%, *v*/*v*) in a −80 °C freezer. All the strains were preserved on GM agar medium.

In order to screen out the strains with nematicidal activity, single colonies were inoculated in GM, NB, and LB liquid media at 28 °C, 180 rpm for 7 days of fermentation, 2 mL of the fermentation solution was spun at 12,000 rpm for 3 min of centrifugation to remove the bacteria, 1.5 mL of supernatant was spread on a plate of 3 cm diameter, about 100–200 *C. elegans* were added for observation, and the experiment was performed in triplicate. The nematode mortality was determined via observation under a light microscope, and nematodes were considered dead if their bodies were immobile, even after mechanical contact. The active fermentation broth was extracted with ethyl acetate, and concentrated and dried under reduced pressure to obtain the crude extract. The extract was dissolved in acetone and diluted with distilled water to reach a final concentration of 1 mg mL^−1^. Approximately 100–200 *C. elegans* were added, then observed and recorded, and the experiment was repeated three times in total.

### 2.4. Identification of Strain WH06

Genomic DNA extraction, polymerase chain reaction amplification, and cloning of the 16S rRNA gene were performed according to the method [23]. An almost complete 16S rRNA gene sequence (1450–1452 bp) was obtained. The sequenced gene sequences were submitted to the NCBI GeneBank database for BLAST comparison analysis with known sequences in the EZBioCloud database (https://www.ezbiocloud.net/) (accessed on 12 July 2021), and the neighbor-joining method of the MEGA-X software was used, and based on 1000 replicates, a phylogenetic tree was constructed.

### 2.5. Fermentation of Strain WH06 and Isolation of Compounds

The conserved strain was inoculated from GM solid medium, and then inoculated on 10 different media (1#–10#) at 28 °C for 10 days to observe the growth of the strain. NB and 1# media were selected to culture the strain WH06 at 28 °C, 180 rpm, and a total of 30 L and 25 L, respectively. The fermentation broth was concentrated under reduced pressure and extracted with ethyl acetate, the total crude extract of NB fermentation broth was 10.33 g and the total crude extract of 1# fermentation broth was 18.03 g.

The extract (10.33 g) from NB fermentation broth was subjected to a Sephadex LH-20 column eluted with methanol to yield six fractions, namely A1~A6. Fraction A4 (5.89 g) was subjected to a Sephadex LH-20 column eluted with methanol to obtain two fractions, A4-1~A4-2. The fraction A4-1 (4.56 g) was submitted to a silica gel column eluted with petroleum ether–acetone (50:1–0:1) to obtain three fractions, A4-1-1~A4-1-3. Fraction A4-1-1 was purified on a Sephadex LH-20 column eluted with chloroform–methanol (1:1, *v*:*v*), and then it was isolated on a silica gel column eluted with chloroform–acetone (100:1–50:1) to obtain compound **1** (7.8 mg). Fraction A4-1-2 (74.6 mg) was isolated using a Sephadex LH-20 column eluted with methanol to produce compound **3** (6.9 mg). Fraction A4-1-3 (168 mg) was submitted to a silica gel column eluted with chloroform–acetone (25:1–1:1) to obtain two fractions A4-1-2-1~A4-1-2-2. Compounds **5** (2.3 mg) and **6** (3.1 mg) were repeatedly purified from A4-1-2-1 (15.5 mg) using a Sephadex LH-20 column eluted with methanol. Fraction A5 (1.19 g) was separated using a silica gel column and eluted with ethyl acetate–acetone (80:1–20:1) to obtain four fractions, A5-1~A5-4. Fraction A5-1 (337.2 mg) was separated on a silica gel column and eluted with petroleum ether–acetone (100:1–20:1) to obtain A5-1-1~A5-1-3. Fraction A5-1-2 was repeatedly purified on a Sephadex LH-20 column eluted with methanol, and then it was submitted to a silica gel column eluted with petroleum ether–ethyl acetate (100:1–20:1) to obtain three parts, A5-1-2-1~A5-1-2-3. Compound **4** (7.4 mg) was obtained via repeated purification from A5-1-2-1 (53.3 mg) using a Sephadex LH-20 column eluted with methanol. Fraction A5-3 (128.6 mg) was purified repeatedly with a Sephadex LH-20 column eluted with chloroform–methanol (1:1, *v*:*v*) to obtain A5-3-2 (14 mg). Fraction A5-3-2 (14 mg) was separated using a silica gel column and eluted with petroleum ether–acetone (10:1–1:1) to obtain two fractions, namely, A5-3-2-1~ A5-3-2-2. A5-3-2-2 (6.2 mg) was purified using a Sephadex LH-20 column eluted with methanol to yield compound **2** (2.3 mg).

The ethyl acetate crude extract (18.03 g) from 1# fermentation broth was submitted to a column of silica gel G (200–300 mesh) and eluted with a petroleum ether–acetone (100:1–0:1) gradient solvent system, followed by ethyl acetate–methanol (10:1–0:1) to yield 10 fractions (B1~B10). B4 (192.1 mg) was subjected to a Sephadex LH-20 column eluted with acetone to yield three fractions B4-1~B4-3. Compound **8** (2.5 mg) was purified repeatedly from B4-1 (29.5 mg) using a Sephadex LH-20 column eluted with methanol and chloroform–methanol (1:1, *v*:*v*). Fraction B4-3 (8.8 mg) was purified using a Sephadex LH-20 column eluted with acetone to produce compound **7** (3.3 mg). B6 (1.36 g) was subjected to a Sephadex LH-20 column eluted with chloroform–methanol (1:1, *v*:*v*) to obtain B6-1~B6-3. B6-3 (145.2 mg) was purified with a Sephadex LH-20 column eluted with methanol to obtain compounds **9** (13.6 mg) and **10** (3.7 mg).

### 2.6. Nematicidal Activities of Isolated Metabolites

The test compounds (**1**–**10**) were dissolved in acetone or methanol, and their nematicidal activities against *M. incognita* were assayed at 400 μg mL^−1^. The same amount of methanol or acetone was used as a blank control, and 150~200 *M. incognita* were added into the solution for observation. All tested compounds were made in three replicates, and the experiments were performed on 24-well plates. Observations were made under the microscope at 24, 48, and 72 h. The active compounds were then assayed at 200, 100, 50, and 25 μg mL^−1^. “Schneider-Orelli formula” corrected mortality (%) = [mortality in treatment group (%) − mortality in control group (%)]/[100 − mortality in control group (%)] × 100 [24].

### 2.7. Test of Inhibitory Activity of Compounds on Egg Hatchings

Four compounds (**1**, **4**, **9**, and **10**) were tested for their inhibitory effects on the egg hatching of *M. incognita*. Egg masses were tested in 24-well plates, with each egg mass placed in a well containing sterile water. The final concentration of the tested compound was 400 μg mL^−1^. The same amount of methanol water was used as the blank control, and all compounds were tested in triplicate. The number of hatching nematodes was recorded after 24, 48, and 72 h. For compounds with an inhibitory effect, three replicates were tested again, and the experimental method was the same as described before.

## 3. Results

### 3.1. Isolation, Identification and Culture of Strain WH06

**Figure 1:**
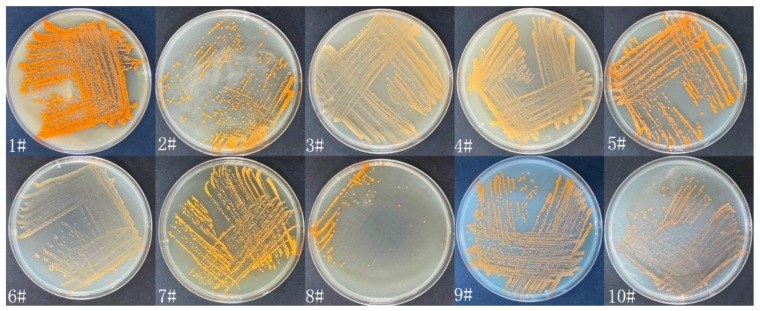
Growth of WH06 cultured on 1#–10# media at 28 °C for 10 days.

Eight strains (WH01-08) of actinomycetes were isolated from soil samples collected in the Tianshan mountains. Among them, the strain WH06 shows nematicidal activity against nematodes. It was cultured on 10 media (1#–10#) to observe its growth status. The growth and morphological characteristics of WH06 cultured for 10 days at 28 °C on 10 different media are shown in Figure 1. It has no aerial mycelium, and its colonies have an yellow–orange color when cultured for 10 days. It grows well on 1# medium.

**Figure 2:**
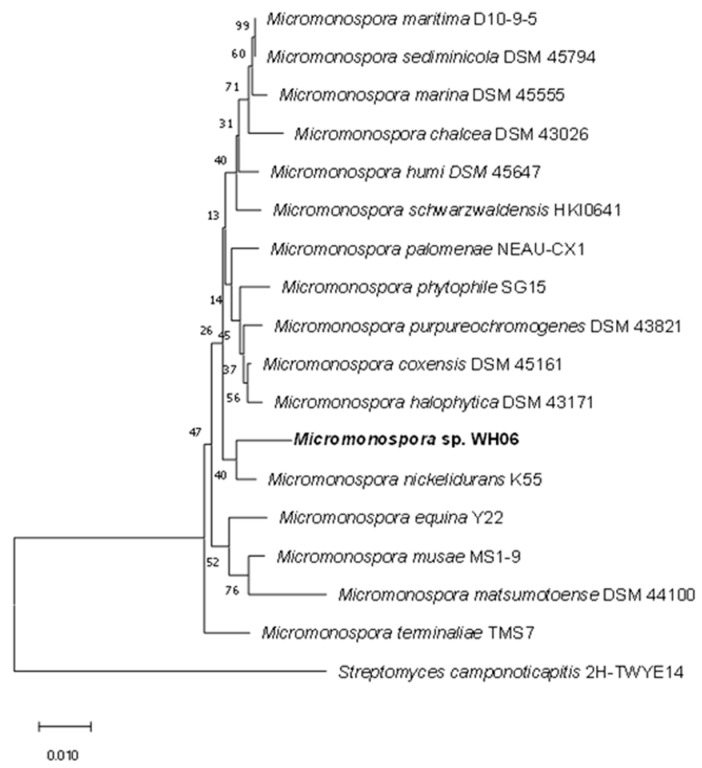
The neighbor-joining phylogenetic tree of *Micromonospora* sp. WH06 on the basis of 1000 replicates.

The strain WH06 was identified via 16S rRNA. It is closely related to *Micromonospora nickelidurans* K55 (98.42%), so it is identified as *Micromonospora* sp. (GeneBank number OP600481). The neighbor-joining method of MEGA-X software was used to construct the phylogenetic tree of *Micromonospora* sp. WH06 on the basis of 1000 replicates (Figure 2).

**Figure 3:**
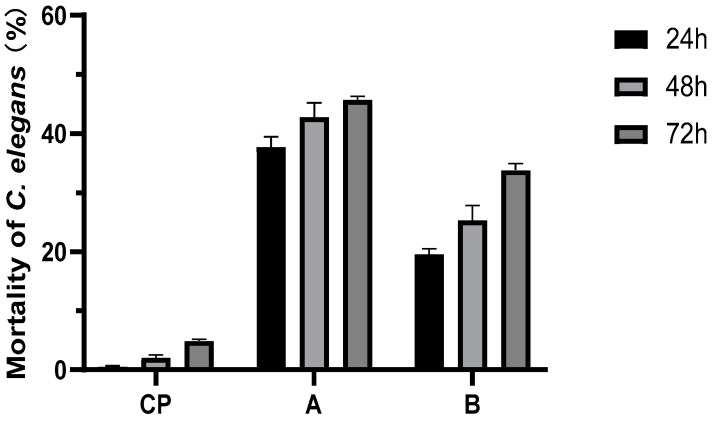
Nematicidal activities of ethyl acetate extracts of strain WH06 cultured in NB and LB media. CP represents blank control with 3% acetone. A represents the ethyl acetate extract of NB fermentation broth, and B represents the ethyl acetate extract of LB fermentation broth. Error bars represent the standard error of the mean (*n* = 3).

*Micromonospora* sp. WH06 shows nematicidal activity against *C. elegans*. At 48 h, the nematicidal activities of broth cultured on NB and LB media cause more than 95% mortality. The GM broth of strain WH06 shows no activity. The extracts of ethyl acetate also cause effective nematicidal activity (Figure 3).

### 3.2. Structural Identification of Compounds

**Figure 4:**
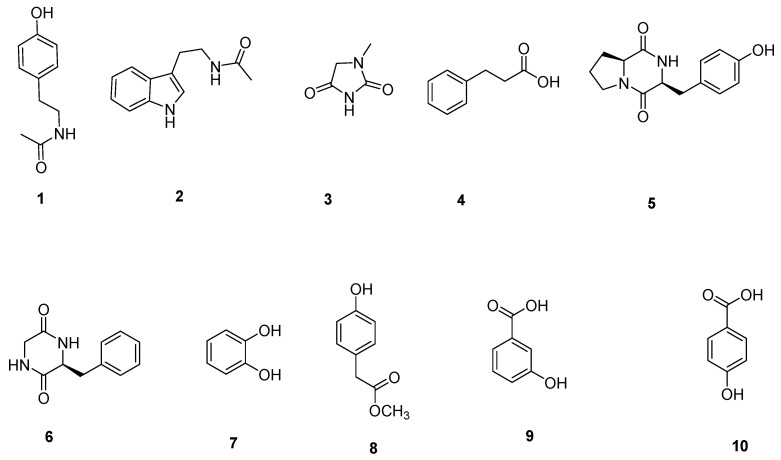
The compounds **1**–**10** isolated from *Micromonospora* sp. WH06.

NB and 1# media were selected to culture *Micromonospora* sp. WH06. Ten compounds (**1**–**10**) were isolated from the ethyl acetate extracts of broth cultured on the two media. Their structures were identified using NMR and MS data (Figure 4).

Compound **1**: yellowish oil; ESI–MS (*m*/*z*): 202 [M + Na]^+^; ^1^H-NMR (600 MHz, CDCl_3_) δ: 7.03 (2H, d, J = 8.3 Hz, H-3/H-5), 6.82 (2H, d, J = 8.3 Hz, H-2/H-6), 3.49 (2H, m, H-8), 2.74 (2H, t, J = 7.0 Hz, H-7), 1.95 (3H, s, H-10); ^13^C-NMR (125 MHz, CDCl_3_) δ: 170.7 (s, C-9), 155.0 (s, C-1), 130.0 (s, C-4), 129.7 (d, C-3/C-5), 115.6 (d, C-2/C-6), 34.6 (t, C-7), 41.0 (t, C-8), 23.3 (q, C-10). It was identified as N-acetyltyramine based on the data of reference [25].

Compound **2**: yellowish solid; ESI–MS (*m*/*z*): 225 [M + Na]^+^; ^1^H-NMR (600 MHz, CDCl_3_) δ: 7.61 (1H, d, J = 7.8 Hz, H-4), 7.40 (1H, d, J = 8.1 Hz, H-7), 7.26 (1H, t, J = 8.1 Hz, H-6), 7.15 (1H, t, J = 7.8 Hz, H-5), 7.06 (1H, s, H-2), 3.61 (2H, t, J = 6.4 Hz, H-11), 3.00 (1H, m, H-10), 1.92 (3H, s, H-4); ^13^C-NMR (125 MHz, CDCl_3_) δ: 170.1 (s, C-13), 136.4 (s, C-8), 127.3 (s, C-9), 122.3 (d, C-7), 122.0 (d, C-2), 119.6 (d, C-5), 118.7 (d, C-4), 113.1 (s, C-3), 111.2 (s, C-7), 39.8 (t, C-11), 25.3 (t, C-10), 23.4 (q, C-14). It was determined to be N-acetyltryptamine based on the data of reference [26].

Compound **3**: white crystal; ESI–MS (*m*/*z*): 115 [M + H]^+^; ^1^H-NMR (600 MHz, CD_3_OD) δ: 3.95 (2H, s, H-5), 2.90 (3H, s, N-CH_3_); ^13^C-NMR (125 MHz, CD_3_OD) δ: 173.8 (s, C-4), 159.3 (s, C-2), 53.9 (t, C-5), 29.2 (q, N-CH_3_). It was determined to be 1-methylhydantoin based on the data of reference [27].

Compound **4**: white crystal; ESI–MS (*m*/*z*): 149 [M − H]^−^; ^1^H-NMR (CDCl_3_, 600 MHz) δ: 7.20–7.31 (5H, m, H-2–H-6), 2.97 (2H, t, J = 7.6 Hz, H-7), 2.69 (2H, t, *J* = 7.6 Hz, H-8); ^13^C-NMR (CDCl_3_, 125 MHz) δ: 178.6 (s, C-9), 140.2 (d, C-1), 128.6 (d, C-3/C-5), 128.3 (d, C-2/C-6), 126.3 (d, C-4), 35.8 (t, C-8), 30.6 (d, C-7). It was identified as benzenepropanoic acid based on the reported data of reference [28].

Compound **5**: white powder; ESI–MS (*m*/*z*): 261 [M + H]^+^; ^1^H-NMR (600 Hz, CD_3_OD) δ: 6.97 (2H, d, J = 8.4 Hz, H-12/H-16), 6.71 (2H, d, J = 8.5 Hz, H-13/H-15), 4.86 (1H, m, H-6), 4.14 (1H, m, H-9), 3.54 (1H, m, H-3a), 3.11 (1H, m, H-3A), 2.88 (1H, m, H-10A), 2.61 (1H, m, H-10B), 2.09 (1H, m, H-5A), 1.91 (1H, m, H-5B), 1.67 (2H, m, H-4); ^13^C-NMR (CD_3_OD, 150 MHz) δ: 171.4 (s, C-7), 167.6 (s, C-1), 158.3 (s, C-14), 132.3 (d, C-12/C-16), 126.9 (s, C-11), 116.4 (d, C-13/C-15), 59.9 (d, C-9), 59.2 (d, C-6), 46.1 (t, C-3), 40.2 (t, C-10), 29.8 (t, C-5), 22.5 (t, C-4). It was identified as cyclo-(L-Pro-L-Tyr) based on the data of reference [29].

Compound **6**: white powder; ESI–MS (*m*/*z*): 205 [M + H]^+^; ^1^H-NMR (600 Hz, CD_3_OD) δ: 7.29 (4H, m, H-9/H-10/H-12/H-13), 7.21 (1H, m, H-11), 4.86 (1H, m, H-6), 4.22 (2H, s, H-3), 3.42 (2H, m, H-7); ^13^C-NMR (125 Hz, CD_3_OD) δ: 170.0 (s, C-1), 168.7 (s, C-4), 136.4 (s, C-8), 131.5 (d, C-10/C-12), 129.6 (d, C-9/C-13), 128.5 (d, C-11), 57.5 (d, C-6), 44.6 (t, C-3), 40.9 (C-7). It was determined to be cyclo(L-Phe-Gly) according to the data of reference [30].

Compound **7**: white powder; ESI–MS *m*/*z*: 109 [M − H]^−^; ^1^H-NMR (600 MHz, CDCl_3_) δ: 6.88 (2H, m, H-3/H-6), 6.83 (2H, m, H-4/H-5); ^13^C-NMR (125 MHz, CDCl_3_) δ: 143.5 (s, C-1/C-2), 121.2 (d, C-4/C-5), 115.5 (d, C-3/C-6). It was identified as catechol based on the spectra data.

Compound **8**: white powder; ESI–MS *m*/*z*: 189 [M + Na]^+^; ^1^H-NMR (600 MHz, CDCl_3_) δ: 7.15 (2H, d, J = 8.4 Hz, H-2/H-6), 6.80 (2H, d, J = 8.4 Hz, H-3/H-5), 3.55 (2H, s, H-7), 3.68 (3H, s, OCH_3_); ^13^C-NMR (125 MHz, CDCl_3_) δ: 172.4 (s, C-8), 154.6 (s, C-4), 130.5 (d, C-2/C-6), 126.2 (s, C-1), 115.4 (d, C-3/C-5), 52.0 (q, OCH_3_), 40.3 (t, C-7). It was identified as methyl (4-hydroxyphenyl) acetate based on the spectra data.

Compound **9**: white powder; ESI–MS *m*/*z*: 137 [M − H]^−^; ^1^H-NMR (600 MHz, CDCl_3_) δ: 7.48 (1H, d, J = 7.7 Hz, H-6), 7.41 (1H, s, H-2), 7.27 (1H, t, J = 7.7 Hz, H-5), 6.99 (1H, d, J = 7.7 Hz, H-4); ^13^C-NMR (125 MHz, CDCl_3_) δ: 169.1 (s, C-7), 158.7 (s, C-3), 133.4 (s, C-1), 130.4 (d, C-5), 121.8 (d, C-6), 120.9 (d, C-4), 117.2 (t, C-2). It was identified as 3-hydroxybenzoic acid based on the spectra data.

Compound **10**: white powder; ESI–MS *m*/*z*: 137 [M − H]^−^; ^1^H-NMR (600 MHz, CDCl_3_) δ: 7.87 (2H, d, J = 8.7 Hz, H-2/H-6), 6.81 (2H, d, J = 8.7 Hz, H-3/H-5); ^13^C-NMR (125 MHz, CDCl_3_) δ: 170.1 (s, C-7), 163.3 (s, C-4), 133.0 (d, C-2/C-6), 122.8 (s, C-1), 116.0 (d, C-3/C5). It was determined to be 4-hydroxybenzoic acid based on the spectra data.

### 3.3. Nematicidal Activities of Compounds

The nematicidal activities of isolated compounds (**1**–**10**) were assayed against the root-knot nematode *M. incognita*. The results show that compound **4** has effective activity against *M. incognita*, causing 100% mortality of *M. incognita* at a concentration of 400 µg mL^−1^ after 72 h. Compounds **1**, **9**, and **10** also have certain toxic effects on *M. incognita* (Table 1). The others show no obvious activity.

**Figure 5:**
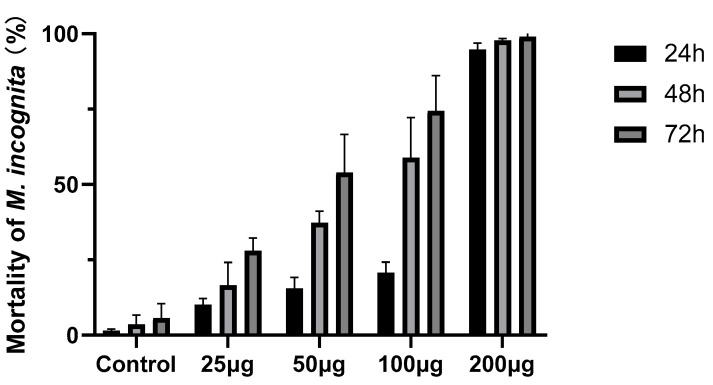
Nematicidal activity of compound **4** against *M. incognita* at different concentrations. Error bars represent the standard error of the mean (*n* = 3).

**Figure 6:**
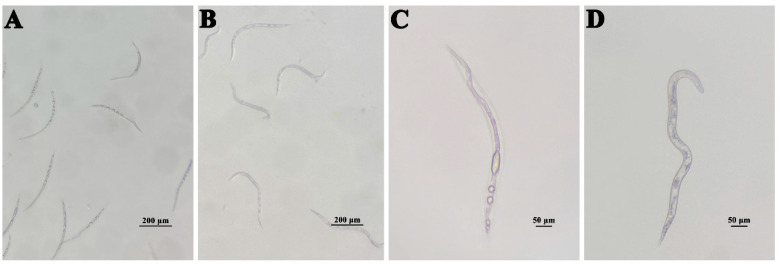
The shapes of active and dead nematodes. (**A**,**C**) *M. incognita* treated with compound **4**. (**B**,**D**) *M. incognita* treated in methanol blank control.

In a further assay, compound **4** was tested against *M. incognita* at different concentrations. The mortality of *M. incognita* reaches 99.02% at 200 µg mL^−1^ at 72 h (Figure 5). The bodies of the dead nematodes treated with compound **4** are slightly curved (Figure 6A), and there are many bubbles in the worms (Figure 6C), while the control nematodes have different morphologies (Figure 6B) and no bubbles are present in the worms (Figure 6D).

### 3.4. Inhibitory Effects of Compounds on Egg Hatching

**Figure 7:**
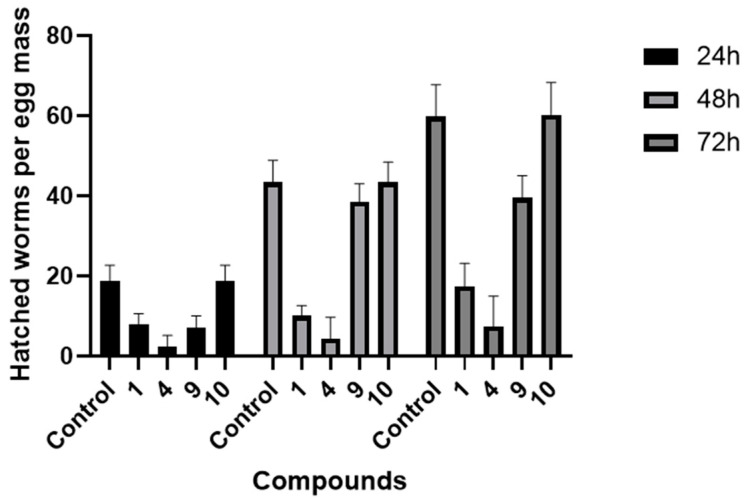
Effects of compounds (**1**, **4**, **9**, and **10**) on the egg hatching of *M. incognita*.

Four nematicidal metabolites (**1**, **4**, **9**, and **10**) were selected to assay their inhibitory effects on the egg hatching of *M. incognita*. The results show that, compared with the control group, compound **4** has a significant inhibitory effect on the egg hatching of *M. incognita* after 72 h of treatment. The number of hatched juveniles per egg is just 7.01. Compound **1** has a weak inhibitory effect, and the other two compounds have no obvious inhibitory activities (Figure 7 and Table S1).

## 4. Discussion

N-acetyltyramine (**1**) is secreted by a new and moderately halophilic actinomycete strain, *Streptomyces* sp. GSB-11, which shows antibacterial activity against certain multidrug-resistant pathogenic bacteria [31]. The compound is also isolated from the marine actinomycete *Streptomyces* sp. KMM 7210, which has antibacterial effects on Gram-positive bacilli and cytotoxic effects on the sea urchin *Strongylocentrotus intermedius* [32]. Compound **1** is obtained from *Actinpolyspora* sp. SIPI-94-1129, and shows inhibitory activity against factor XIIIa in the range of 0.2~12.8 mM [33]. In addition, compound **1** is purified from *Microbispora aerata* IMBAS-11 isolated from Antarctic penguin feces [34].

N-acetyltryptamine (**2**) is isolated from *Streptomyces djakartensis* NW35 [35] as well as *Streptomyces* sp. strain TN58 [36], and it has significant antibacterial activity [35]. Compound **2** is also obtained from the marine fungus, *Gracilaria verrucosa* [26]. In addition, it is purified from the marine bacterium *Roseivirga echinicomitans* KMM 6058^T^, and shows cytotoxic activity against mouse erythrocytes [37]. The compound is shown to antagonize the inhibitory effect of melatonin on the release of [^3^H] dopamine from the retina [38].

Benzenepropanoic acid (**4**) is generally used as a pharmaceutical intermediate and also in organic synthesis. In this paper, its nematicidal activity against *M. incognita* is reported for the first time, which can help with the development of new potential pesticides.

Cyclo-(L-Pro-L-Tyr) (**5**) exhibits growth inhibitory activity against the newly hatched larvae of *Helicoverpa armigera* (Hubner), with IC_50_ values of 50~200 μg mL^−1^ [39]. A structural analogue of **5**, cyclo-(Pro-Tyr), is toxic and selective against MCF-7 cells isolated from *Barrientosiimonas humi* [40], and has potential anticancer effects on N-diethyl-nitrosamine-induced hepatocellular carcinoma in mice via the PI3K/AKT signaling pathway [41]. Another structural analogue, cyclo-(L-Pro-D-Tyr), is isolated from *Streptomyces* sp. strain TN58 and exhibits some antibacterial activity [42]; it is also obtained from Huangjing wine and exhibits antioxidant activity in DPPH (2,2-diphenyl-1-picrylhydrazyl) [43]. In addition, cyclo-(D-Pro-L-Leu) produced by *Bacillus amyloliquefaciens* Y1 is identified for the first time as a nematicide for the control of *M. incognita* [44]. However, in this study, compounds **5** and **6** do not show significant nematicidal activities.

4-Hydroxybenzoic acid (**10**) has antibacterial, antialgal, antimutagenic, antiestrogenic, hypoglycemic, anti-inflammatory, anti-platelet aggregation, nematicidal, antiviral, and antioxidant properties and can be used as a preservative [45]. It has significant nematicidal activity against root-knot nematodes and *Cephalobus litoral* [46], and the EC_50_ against *M. incognita* at 48 h is 871 μg mL^−1^ [47]. The 3-Hydroxybenzoic acid (**9**) also shows some nematicidal activity, but not as much as compound **10**. A class of these compounds has been shown to have nematicidal activity, such as 3,4-dihydroxybenzoic acid [48], 3-methoxy-4-hydroxybenzoic acid [49], 5 dihydroxy benzoic acid, and gallic acid [50].

In this paper, we report for the first time the root-knot nematicidal as well as egg hatching inhibitory activities of strains of *Micromonospora* sp. Root-knot-nematode-infested plants are susceptible to secondary infestation by phytopathogenic bacteria, which may have additional detrimental effects on plant growth, and cause more damage [3]. Nematicidal actinomycetes are proven to be antagonistic to other plant pathogens, including fungal and bacterial organisms [51]. Several studies show that *Micromonospora* species play an important role in biological control, plant growth promotion, and rhizosphere ecology [52]. Therefore, compounds with the bacteriostatic activity of this *Micromonospora* sp. can cooperate with nematicidal compounds to kill root-knot nematodes while inhibiting the secondary infestations of other pathogenic bacteria, thus, positively affecting plants. *Micromonospora* sp. WH06 is a potent strain of biocontrol for the root-knot nematode.

Although natural products have potential applications in the biocontrol of nematodes, they also have several limitations. The cost of preparing nematicidal metabolites must be considered, and some of them are limited because of their low production. Some nematicidal metabolites exhibit high effects in vitro against nematodes, but they show low activity in the soil environments. At the same time, they may change soil environmental pH and regulate soil microenvironments. So, it is important to test the effectiveness of active products during practical application by pot or field trials.

## Figures and Tables

**Table 1:** The nematicidal activities of compounds against *M. incognita*.

Compounds	Mortality of *M. incognita* (%) ± SD		
	24 h	48 h	72 h
(**1**)	9.06 ± 2.42	10.05 ± 2.43	36.43 ± 4.89
(**4**)	89.68 ± 2.91	98.46 ± 1.43	100 ± 0
(**9**)	29.10 ± 5.15	30.32 ± 4.13	40.75 ± 5.26
(**10**)	22.33 ± 5.40	40.19 ± 4.24	62.10 ± 5.37
Control	0.65 ± 0.20	3.62 ± 0.98	5.59 ± 1.28

The numbers represent average ± standard error from three replicates.

## Data Availability

Not applicable.

## Supplementary Materials

The following supporting information can be downloaded at: https://www.mdpi.com/article/10.3390/microorganisms10112274/s1.
