# Supplementary material for: Nematicidal Metabolites from the Actinomycete Micromonospora sp. WH06
Source: Microorganisms. 2022 Nov 16;10(11):2274. doi: 10.3390/microorganisms10112274 (PMC9693860; doi:10.3390/microorganisms10112274)
Supplement: Supplementary file 1 [file microorganisms-10-02274-s001.zip › microorganisms-2021156-supplementary.pdf]

Supplementary Materials for

Nematicidal Metabolites from the Actinomycete *Micromonospora* sp. WH06

Yuan Ran, Yu Zhang, Xin Wang* and Guohong Li*

State Key Laboratory for Conservation and Utilization of Bio-Resources in Yunnan, and Key Laboratory for Southwest Microbial Diversity of the Ministry of Education, Yunnan University, Kunming, 650091, China
* Correspondence: ligh@ynu.edu.cn (L.G.)

Table S1. Effects of compounds (**1**, **4**, **9**, and **10**) on the egg hatching of *M. incognita.*

**Compounds**	**Hatched worms per egg mass ± SD**		
	24h	48h	72h
(**1**)	8.00±2.65	10.00±2.64	17.33±5.86
(**4**)*	2.33±2.91	4.34±1.00	7.01±5.57
(**9**)	7.00±4.24	38.50±4.95	39.50±4.95
(**10**)	19.33±4.24	42.67±9.29	60.33±4.51
Control	18.71±3.61	43.40±4.75	59.80±6.50

* Mean ± SD of 6 replicates
